# Evaluating of Physiological Chemical Levels in Blood to Assess the Risk of Morbidity and Mortality of Ischemic Cardiovascular Disease

**DOI:** 10.3390/ijerph120911549

**Published:** 2015-09-14

**Authors:** Junyan Teng, Yanping Wei, Fengming Su, Zhiping Guo, Jing-Quan Zhong

**Affiliations:** 1 The Key Laboratory of Cardiovascular Remodeling and Function Research, Chinese Ministry of Education and Chinese Ministry of Health; The State and Shandong Province Joint Key Laboratory of Translational Cardiovascular Medicine, Qilu Hospital of Shandong University, Jinan 250012 , Shandong, China; E-Mail: junyanteng@hotmail.com; 2 Luoyang Orthopedic-Traumatological Hospital, Zhengzhou 450000, Henan, China; E-Mail: guozhiping74@hotmail.com; 3 Henan Province People’s Hospital, Zhengzhou 450003, Henan, China; E-Mail: wei_yp1234@163.com; 4 Emergency Medical Rescue Center of Zhengzhou City, Zhengzhou 450000, Henan, China; E-Mail: zz120su@163.com

**Keywords:** physiological chemicals, risk score, morbidity and mortality, hepatic disorder, cardiac disorder

## Abstract

In this study, a multiple linear regression model to evaluate the risk of morbidity and mortality of ischemic cardiovascular disease is demonstrated. In this model, predictor variables are selected from physiological chemicals in a blood test of the subjects. Meanwhile, the calculated risk score is selected as a response variable. Four major latent variables including hepatic, nephric, metabolic, and BMI (Body Mass Index) are revealed by performing statistical and principal component analysis for the collected survey data. The analyzed result also shows that the cardiac disorder is correlated with symptoms of abnormal BMI, hepatic disorder, nephric disorder, and metabolic disorder. Thus, the risk of morbidity and mortality of ischemic cardiovascular disease can be assessed from the proposed multiple regression model.

## 1. Introduction

Cardiovascular disease is one of the leading causes of death [1]. From an epidemiology point of view, if some of the levels of physiological chemicals (markers) in blood could reveal the possibility of cardiac disorder, the death rate of Ischemic Cardiovascular Disease (ICVD) should be reduced. The laboratory blood test is a tool helpful in evaluating the health status of an individual [2]. For instance, the level of lactate dehydrogenase (LDH) in blood rises when the patient is suffering from myocardial infarction, pulmonary embolism, hepatic disorder, and muscle disorder [3]. Blood creatinine kinase (CK) is a cardiac predictive marker. Creatine Kinase (CK) is the acute cardiac injury flag because of its excellent specificity. The ratio of CK-MB to total CK, especially when the CK-MB rises, is a sensitive indicator of myocardial injury [4,5].

Previous reports pointed out that a 1 mmol/L increase in the plasma triglyceride level might cause a two-fold increase in the risk of cardiovascular disease for the male gender [6]. However, even though blood triglycerides (TG) are an important factor that affect the risk of cardiac disorder, no direct evidences shows that the increasing triglyceride level is an independent predictor for cardiovascular disorder [7].

Serum alkaline phosphatase (ALP) plays a physiological role in affecting the risk of cardiovascular disorder [8,9]. ALP subjects physiological variations are closely to associated with age based upon the physiological hormonal changes. As found in human blood of hepatic disease without jaundice, ALP elevation alerts the risk of cardiac disorder. We are not surprised that the presence of the predictive marker of hepatic disorder is highly correlated with cardiovascular disease [10].

Alanine aminotransferase (ALT), also called SGPT (serum glutamic-pyruvic transaminase) or GPT, is an enzyme indicator in blood. The level of ALT is generally less than 30−40 U/L. ALT rises to indicate the hepatic damage. AST (aspartate aminotransferase) is called SGOT (serum glutamic-oxalocetic transaminase) or GOT. The blood test level of AST is generally less than 40–50 U/L. ALT and AST are the ones used to test liver function; however, in the heart, tissue damage can also result in increasing blood ALT [11].

With respect to the ALT, AST is more non-specific. Increased AST is an important indicator of hepatic injury [12]. Physicians have been interested in if GPT or GOT affects the cardiac disorder for decades [13]. In China, based on 17 years of follow-up observation and using of the Cox proportional hazard model to evaluate the risk of morbidity and mortality of cardiac disorder, risk score (RS) calculation procedures was reported by Wu *et al.* [14]. However, we concern about if the ICVD can be found in a head of time via the precursors in the blood. Through the RS in terms of the markers in blood, we might be able to assess and alert the clinicians to the risk of ICVD in the patients [15].

In RS calculation procedures, the age, systolic blood pressure (SBP), body mass index (BMI), serum total cholesterol (TC), diabetes (GLU), and smoking history were the six factors collected as independent (predictor) variables. To explain the features of RS, we considered a 50-year-old male subject with a systolic blood pressure of 150/90 mmHg, BMI of 25, serum total cholesterol of 5.46 mmol/L, and positive smoking history but no diabetes as an example to calculate the RS for assessing the risk of morbidity and mortality of ICVD. Following the standardizations by Wu *et al.* [14], parameter X1 represents the age effect; because the subject is 50 years old, X1 is assessed at three points for estimating the risk of morbidity and mortality of ICVD. Parameter X2 represents the blood pressure effect, and the systolic blood pressure of the subject is 150 mmHg, so X2 is assessed at two points for the risk estimation. Parameter X3 represents the BMI effect and subject has a BMI of 25, so X3 is assessed at only one point toward the risk of morbidity and mortality related to ICVD. X4 represents the total cholesterol (TC) effect and the level in the blood of the subject is 5.46 mmol/L, so X4 is assessed to be one point. X5 represents the subject’s lifestyle; smoking is assessed at two points (no smoking is assigned zero points). X6 represents the subject’s lack of diabetes, and it is assessed at zero points for the risk (with diabetes assessed as one point). Summing the points from X1 to X6, 3 + 2 + 1 + 1 + 2 + 0 = 9, we can get the risk score of nine points, which means the subject has a low risk level of ICVD under the criteria of the safe level (RS < 8), low risk level (8 ≤ RS ≤ 10), and high risk level (RS > 10). Equation (1) demonstrates the calculation of RS proposed by Wu *et al.* [14]

(1) RS = X1 + X2 + X3 + X4 + X5 + X6

In this study, collecting enough subjects from the general population in Henan, China, we want to calculate RS for the subjects. Meanwhile, we assume that the RS is correlated with the selected predictors in blood so that we may find the significance of the markers in the blood to assess the risk of morbidity and mortality of ICVD statistically. By using principal component analysis (PCA), four principal predictors were revealed, and they are hepatic-, nephritic-, metabolic-, and BMI-related markers. The hepatic (H)-related markers comprise ALT, GOT, and GGT. The metabolic (M)-related markers include alkaline phosphatase (ALP), low-density (LDL) and high-density cholesterol (HDL), and triglycerides (TG). The nephritic (N)-related markers comprise creatine (CR) and uric acid (UA). Originally, with BMI (B), 10 predictors were selected for a linear regression to predict a response variable RS. The significance of those factors’ influence on the risk of morbidity and mortality of ICVD were reviewed. Meanwhile, a new predictive model for RS in terms of H, M, N, and B to assess the risk of morbidity and mortality of ICVD were proposed and analyzed.

## 2. Methods

### Design and Sample

Since three million people die with ICVD in China each year, the research of the associations on the morbidity and mortality of ICVD has become an important concern [14]. There were 2400 cases where the subjects, male or female, ranging 35 to 59 years old, were assigned randomly to answer specific medical questionnaires as well as to do lab blood tests. The aim of this study is to specify the degree of significance of some specific hepatic enzymes, serum cholesterol, metabolic physiological chemicals, and TG level effects presented by RS in the blood to evaluate the risk of morbidity and mortality of ICVD. In previous research, six predictor variables included age, systolic blood pressure (SBP), body mass index (BMI), serum total cholesterol (TC), with or without diabetes (GLU), and smoking history, and these were well defined to calculate RS. In this study, we stratified the RS into three levels comprising the safe level (RS < 8), the low risk level (8 ≤ RS ≤ 10), and the high risk level (RS > 10). The former research revealed that RS for evaluating the risk of the morbidity and mortality of ICVD is significantly associated with gender, age, blood pressure, body mass index, smoking, and diabetes mellitus. However, the association of the physiological chemicals in blood with response variable RS to assess the risk of morbidity and mortality of ICVD was not involved in the previous study [13,14,15].

In order to get the response variable RS in terms of the predictor variables of BMI, LDL, HDL, GOT, GGT, ALT, ALP, TG, CK, and uric acid (UA) for assessing the risk of morbidity and mortality of ICVD, we used multiple regression procedures to create a regression model to calculate the RS with those predictors. We attempt to understand the physiological chemical levels in the blood comprising ALT, GOT and GGT, TG, low-density cholesterol, high-density cholesterol, creatinine, and ALP correlated with the risk of morbidity and mortality related to ICVD.

As we planned, 2400 subjects in Henan Provincial People’s Hospital, aged from 35 years old to 59 years old, were collected from physical health lab. Of the 2400 subjects, were corresponded with. The selected effective rate was 92.13%. Survey information included demographic data (gender, age, nature, and position of units), lifestyle (smoking and drinking), medical history (history of diabetes mellitus, coronary artery disease, and cerebral apoplexy), and the record of physical examination (systolic blood pressure, total cholesterol, and body mass index), *etc.* Blood lipid and blood glucose levels were all checked using Switzerland Roche reagents (Roche Diagnostics GmbH., SandhoferStrasse 116, 68,305 Mannheim, Germany) for calculating RS. Meanwhile, we selected and measured the levels of BMI, ALT, GOT, GGT, TG, LDL, HDL, CK, UA, and ALP from the blood test to associate with RS by multiple-variable regression procedures as well as principal component analysis.

## 3. Statistical Analysis and Multiple-Variable Regression Procedures

From the data of the blood test, we realized that none of the physiological chemicals could be an independent affecting factor for assessing the risk of cardiac disorder. We realized that, for male gender, the coefficients of correlation between RS and each of the two variables could not be in linearity because the *p*-value for testing was not significant (*p >* 0.001). For female subjects (not shown), the significance of the testing should not be able to reject the null hypothesis (no linear correlation being found), which means the coefficient of the correlation is close to zero. Thus, we decided to stratify the RS and use principal component analysis (PCA) to find the significance of the predictor variables and then perform a multiple-variable regression analysis to calculate a response variable RS in terms of predictor variables for assessing the risk of morbidity and mortality of ICVD for the subjects without gender classification.

By using principal component analysis, we obtained four principal components comprised of H (Hepatic), N (Nephritic), M (Metabolic), and B (BMI). Using a step-by-step approach, we first correlated nine different observation variables including hepatic enzymes ALT, GOT and GGT, TG, low-density cholesterol, high-density cholesterol, CK, UA, and ALP. Then, we performed statistical analysis to get the weighting coefficient for each principal component associated with the response variable (RS) to assess the risk of morbidity and mortality of ICVD. Finally, a multiple-variable regression analysis was used to reveal the rank of significance of the observations at different stratification levels of RS [16] and, then, we completed a model with a good coefficient of the determination.

## 4. Results

In Table 1, it shows the Pearson’s correlation coefficients and the p-value for testing the significance of the selected physiological chemicals (marks) in blood.

**Table 1:** Pearson’s Correlation Coefficients between risk scores (RS) and the markers. Significance test *p* < 0.001 is required to reject Null hypothesis. Null hypothesis means the linear correlation is not existed at the significance *p*-value that *p* > 0.001.

	BMI	HDL	LDL	TG	CK	UA	ALP	ALT	GOT	GGT
A **	0.481	(0.111) *	0.300	0.292	0.241	0.220	0.187	0.191	0.127	0.286
B **	0.000	0.000	0.000	0.000	0.000	0.000	0.000	0.000	0.000	0.000
C **	0.177	0.033	0.038	0.110	0.074	(0.140) *	0.145	0.125	0.138	0.253
D **	0.000	0.000	0.000	0.000	0.000	0.000	0.000	0.000	0.000	0.000
E **	0.385	0.040	0.192	0.224	−0.011	0.071	0.099	0.085	0.058	0.237
F **	0.000	0.063	0.000	0.000	0.334	0.004	0.000	0.001	0.013	0.000

* Negative Numbers; ** A: all RS selected (include both male and female); B: *p*-value for significance (one-tailed) test of A; C: data selected only at RS = 3 (include both male and female); D: *p*-value for significance (one-tailed) test of C; E: all RS selected for male subjects only; F: *p*-value for significance (one-tailed) test of E.

Table 2 shows the abnormality of the levels of the predictors in the blood. In this study, we calculated and used RS of the subjects to evaluate the risk of morbidity and mortality of ICVD. Meanwhile, we also used the software IBM SPSS 22 to implement the statistical data analysis and principal component analysis to construct a multiple regression model.

**Table 2:** Abnormal level of the selected markers in blood.

Markers	Level
BMI	25
HDL/high density lipoprotein	>1.8 mmol/L
LDL/low density lipoprotein	>3.36 mmol/L
TG/triglyceride	>2.25 mmole/L
CK/creatininekinase	male > 104 μmol/L
	female > 84 μmol/L
Uric Acid	male > 416 μmol/L
	female > 257 μmol/L
ALP/alkaline phosphatase	>129 U/L
GOT/oxaloacetic transaminase	>33 U/L
GGT/glutamyltranspeptidase	>40 U/L
ALT/alanine Aminotransferase	>81 U/L

The RS was stratified into three levels as no risk (RS = 1), low risk (RS =2), and high risk (RS = 3). Performing the PCA and multiple regression analysis, we reduced the predictor variables to get four mixed PCA predictor variables. Based upon our analysis, at different levels of stratified RS, the form of the proposed multiple-variable regression model is presented as Equation (2).

In Table 3, calculated β coefficients correlated to the predictor variables for the response of RS are presented. Predictor variables include ASP, ALT, GGT, GOT, TG, CK, UA, LDL, HDL, and BMI, and we used them to calculate the response RS and construct a multiple regression model with the linear combination of those predictor variables. Here we named it RM1.

(2) RS = β_0_ + β_1_H + β_2_N + β_3_M + β_4_B

**Table 3:** RM1 multiple-variable regression model of the selected markers in blood for the clinical population collected in Henan Province, China.

RM1: RS	Coefficients without Standardization		Standardized Coefficient	*t*	Significance *p*-Value	95.0% Confidence Interval of β	
	Estimation of β	Standard Error	Beta Distribution			Lower Bound	Upper Bound
Constant	−12.714	0.749		−16.973	0.000	−14.183	−11.245
BMI	0.409	0.022	0.389	18.980	0.000	0.366	0.451
HDL	0.585	0.232	0.049	2.519	0.012	0.130	1.041
LDL	0.801	0.086	0.173	9.310	0.000	0.632	0.970
TG	0.227	0.044	0.100	5.134	0.000	0.140	0.314
CK	0.020	0.005	0.082	4.184	0.000	0.011	0.030
UA	0.248	0.055	0.085	4.488	0.000	0.140	0.356
ALP	0.011	0.003	0.070	3.717	0.000	0.005	0.016
ALT	−0.014	0.006	−0.081	−2.429	0.015	−0.025	−0.003
GOT	0.013	0.011	0.039	1.215	0.225	−0.008	0.034
GGT	0.011	0.002	0.113	5.317	0.000	0.007	0.015

The multiple-regression model RM1 presented in Table 3 is presented in Equation (3)

(3) RS = −12.714 + 0.409 × BMI + 0.585 × HDL + 0.801× LDL + 0.227 × TG + 0.020 × CR + 0.248 × UA + 0.011 × ALP − 0.014 × ALT + 0.013 × GOT + 0.011 × GGT

In Table 4 and from Equation (4) to Equation (7), we show the impact weighting coefficients of the principal components analysis, which were proceeded by using the software IBM SPSS 22. The presented impact weighting coefficient for each component was calculated at a specific risk value where GOT, GGT, CR, UA, TG, and CK are the levels of the selected markers in the blood collected from the subjects.

(4) H = H_11_ × GOT + H_12_ × GGT

where H_11_ = 0.808 and H_12_ = 0.750 are the impact weighting coefficients

(5) N = N_11_ × CR + N_12_ × UA

where N_11_ = 0.737 and N_12_ = 0.598 are the impact weighting coefficients

(6) M = M_11_ × TG + M_12_ × CK

where M_11_ = 0.737 and M_12_ = 0.598 are the impact weighting coefficients

(7) BMI = Body weight (Kg)/Square of the Height of the body (m^2^).

These four integrated factors, H, N, M, and B, affect the risk of morbidity and mortality related to ICVD. By using them, we proposed a multiple-variable regression model of RM2.

**Table 4:** Impact weighting coefficients of the principal components presented by the selected physiological chemicals (markers) in blood for a specific RS value.

	H	B	N	M
BMI	0.182	0.744	−0.009	−0.003
HDL	0.494	−0.196	−0.158	−0.302
LDL	0.329	0.482	0.141	−0.520
TG	0.227	0.544	−0.168	0.221
CK	−0.216	0.116	0.737	0.358
UA	−0.289	0.369	0.598	−0.224
ALP	0.193	0.480	−0.277	−0.143
ALT	0.746	−0.109	0.475	0.017
GOT	0.808	−0.325	0.343	−0.089
GGT	0.750	−0.031	−0.198	0.176

Table 5 shows the β coefficients correlated to the predictor variables for the response RS presented by the linear combination of H, B, N, and M, the four principal components. From the analysis, the sort order of the significance of the principal components affecting the risk score is H > B > N > M when RS is equal to 3 (RS = 3). Meanwhile, H > M > N > B is the order when RS is equal to 2 (RS = 2). Equation (8) is the multiple-variable regression model of RM2. Based upon the analysis results, we realized that the significance of the components is varied via the level of risk score to affect the assessment of the risk of ICVD.

(8) RS = 11.417 + 0.222 × H − 0.101 × N + 1.914 × M + 0.425 × B

**Table 5:** RM2 multiple regression model coefficients of the principal components of the correlation matrix of the selected physiological chemicals in the blood of subjects.

RM2: RS	Coefficients without Standardization		Standardized Coefficient	*t*	Significance *p*-Value	95.0% Confidence Interval of β	
	Estimation of β	Standard Error	Beta Distribution			Lower Bound	Upper Bound
Constant	11.417	0.062	--	185.305	0.000	11.296	11.538
Hepatic	0.222	0.041	0.042	5.398	0.000	0.141	0.303
BMI	0.425	0.030	0.123	14.168	0.000	0.366	0.484
Nephric	−0.101	0.029	−0.031	−3.494	0.000	−0.157	−0.044
Metabolic	1.914	0.020	0.886	96.382	0.000	1.875	1.953

Comparing the two multiple-variable regression models RM1 and RM2, we realized that the determination coefficients increased from 0.336 to 0.878 after principal component analysis (PCA) proceeded. With an orthogonal transformation converting a set of predictor variables to a projected set, a set of weighting numbers for uncorrelated variables can be assigned to linearize correlations for predictor variables and the response variable. Table 5 also shows the weighting numbers of the four major components H, N, M, and B that contributed to the response variable. Comparing Equation (3) and Equation (8), we realized that the predictor variables are reduced to four major components by the principal component analysis. To calculate RS in terms of H, N, M, and B, it would be the same as calculating RS in terms of X1, X2, X3, X4, X5, and X6. Multiple-variable regression proceeds with different predictors, and both H, N, M, and B, and X1, X2, X3, X4, X5, and X6 should be at the same level of RS at a reasonable determination coefficient.

Table 6 shows the determination coefficients of the models RM1 and RM2. Comparatively, we realized that in the RM2 model, the Durbin-Watson test is larger than 1.5 (criteria value) and the coefficient of the determination (CD) is 0.8 and the correlation coefficient reaches 0.937 (higher is better), indicating a good model fit to the statistic criteria to assess the risk of morbidity and mortality of ICVD presented by the risk score.

**Table 6:** Coefficients of determination (CD).

	R	R Square (CD)	Standard Error of the Estimation	Durbin-Watson Test
RM1	0.580	0.336	2.726	1.359
RM2	0.937	0.878	1.169	1.764

## 5. Discussions

Based upon causality theory, the independent predictor is an only factor that can cause a specific effect. In this study, no single predictor was revealed to affect the risk of morbidity and mortality of ICVD alone. Thus, we conclude that none of the markers we discussed can be an independent predictor. A multiple linear regression model is thus proposed to present the scale of contribution of every marker that contributes to a risk score. We selected several markers to be the predictor variables, including ALT, ASP, GOT, GGT, LDL, CK, TG, UA, and BMI. In this study, we realized all of our selected markers are responsible for affecting the risk of morbidity and mortality related to ICVD.

Typical methods to estimate risks of cardiovascular disease includes the Cardiovascular Disease Risk Calculator proposed by Kaplan and Meier [17], Cox’s proportional hazards model [18], Canadian [19] and American Heart Association (AHA) [20] guidelines, *etc.* However, those methods are for clinicians who consider risk factor burden in the patients’ lifetime a risk for cardiovascular disease. In our study, we proposed an easy and accurate multiple-variable regression model for all populations that do and do not have specific cardiovascular disease symptoms. With the data of regular lab blood tests and our proposed model, people can get the risk score to assess their risk for ICVD. It will be helpful for the populations who want to find out their risk of morbidity and mortality of ICVD when they do regular health exams.

Basically, the existing models referred to in the references [17,18,19,20] are not really applicable for causality analysis in etiology, which means those existing models and knowledge are not able to explain which factors affect the risk of morbidity and mortality of ICVD. In this study, we present our model from an epidemiological point of view, ending with integrated hepatic, nephric, metabolic, and BMI-related predicators which may correlate to the etiological point of view to explain the factors that affect the risk of morbidity and mortality related to ICVD.

Based upon the ICVD risk score computing procedures proposed by Wu *et al.* in 2003 [14], the risk factors comprise age, SBP, BMI, serum total cholesterol (TC), presence of diabetes (GLU), and smoking history. Then, we stratified the RS into several levels to show the different significance of the impact of the markers for evaluating the risk of morbidity and mortality of ICVD. The principal component analysis (PCA) of the correlation matrix of selected markers from the subjects revealed four major factors which are hepatic (H), metabolic (M), nephritic (N), and BMI (B). The rank order of the major components presents the significance level of the impact of those factors. H > B > N > M is the order when the RS is equal to 3 (RS = 3), but when the RS equals 2, the significance level of the impact that affects evaluating the risk of ICVD is changed to H > M > N > B for the subjects. Furthermore, GOT, GGT, CK, and TG were realized to be the top four important markers in the blood that affect the risk of morbidity and mortality related to ICVD.

Statistically, the proposed multiple-variable regression model for calculating RS in terms of the four major principal components H, M, N, and B has shown the linear combinations of H, M, N, and B with an acceptable coefficient of determination as well as the weighting coefficients. From the epidemiological point of view, our study is to note that clinicians should remind their patients to pay full attention to their lab blood test results. Any high hepatic, nephritic, or metabolic levels and quick BMI changes mean the body is providing a warning of the risk of morbidity and mortality related to ICVD.

## 6. Conclusions

An independent predictor means that only one causal factor exists to initiate a specific effect [21]. In this study, no single independent predictor was revealed, which means none of the markers we discussed can be an independent predictor that affects the risk of ICVD. A linear multiple-variable regression model is thus proposed to present the substantial function of each marker that we discussed. The calculated weighting coefficients in the model can present the probability distribution to the calculated RS proposed by Wu *et al.* [14], which is also correlated with Cox’s proportional hazards model to assess the risk of morbidity and mortality of ICVD. By using PCA for the data analysis, it is revealed that the H, N, M, and B impact the RS to assess the risk of morbidity and mortality of ICVD for the clinical population selected. Statistically, good CD, R values, and a Durbin-Watson Test can guarantee a good model for the selected population. Meanwhile, RS is also recognized as a good index to assess the risk of morbidity and mortality of ICVD. Therefore, we conclude that our proposed model is valuable for using physiological chemicals (markers) in the blood to assess the risk of morbidity and mortality of ICVD.
